# Nonlocal Buckling Analysis of Composite Curved Beams Reinforced with Functionally Graded Carbon Nanotubes

**DOI:** 10.3390/molecules24152750

**Published:** 2019-07-29

**Authors:** Behrouz Karami, Maziar Janghorban, Davood Shahsavari, Rossana Dimitri, Francesco Tornabene

**Affiliations:** 1 Department of Mechanical Engineering, Marvdasht Branch, Islamic Azad University, Marvdasht 73711-13119, Iran; 2 Department of Innovation Engineering, University of Salento, 73100 Lecce, Italy

**Keywords:** buckling, Galerkin method, nanocomposites, nonlocal elasticity theory

## Abstract

This work deals with the size-dependent buckling response of functionally graded carbon nanotube-reinforced composite (FG-CNTRC) (FG-CNTRC) curved beams based on a higher-order shear deformation beam theory in conjunction with the Eringen Nonlocal Differential Model (ENDM). The material properties were estimated using the rule of mixtures. The Hamiltonian principle was employed to derive the governing equations of the problem which were, in turn, solved via the Galerkin method to obtain the critical buckling load of FG-CNTRC curved beams with different boundary conditions. A detailed parametric study was carried out to investigate the influence of the nonlocal parameter, CNTs volume fraction, opening angle, slenderness ratio, and boundary conditions on the mechanical buckling characteristics of FG-CNTRC curved beams. A large parametric investigation was performed on the mechanical buckling behavior of FG-CNTRC curved beams, which included different CNT distribution schemes, as useful for design purposes in many practical engineering applications.

## 1. Introduction

The reinforcement of nanocomposites with the introduction of carbon nanotubes (CNTs) as filler beside a polymeric matrix is well known to improve the potential applications of a structure in some fields of mechanics and electronics. Indeed, in recent decades, CNTs reinforced nanocomposites have been increasingly studied in the scientific community because of their remarkable properties [1,2,3,4,5,6,7,8,9]. CNTs are made of graphene sheets as it is the thinnest material in the world. Therefore, the use of CNTs with very small dimensions cannot disregard the possibility of size-dependent behavior of materials, especially at a nanoscale. This represents a challenging aspect to consider during the evaluation of the structural behavior of nanomaterials. To overcome this issue, a large variety of methods and strategies have been proposed in the literature, including laboratory tests, molecular dynamics-based simulations, and non-classical mathematical methods [10,11,12,13,14,15,16,17,18,19]. Among them, experimental tests and molecular dynamics simulations, however, are typically expensive and time-consuming, which has led to find an attention to use theoretical and numerical models for approaching similar problems. In this framework, Eringen [20,21] proposed a size-dependent model in which the size-dependent behavior is considered by introducing one small-scale nonlocal parameter. However, this approach considers only the softening enhancement of the size-dependence in nanostructured systems. Bouafia et al. [22] analyzed the bending and vibration response of FG nanobeams via a nonlocal quasi-3D theory. Shahsavari et al. [23] studied the forced vibration of viscoelastic graphene sheet under the moving load using a nonlocal refined plate theory. Ganapathi et al. [24] studied the vibrations of curved nanobeams via a nonlocal higher-order theory based on a finite element approach. For the first time, a guided wave propagation analysis of porous nanoplates was performed by Karami et al. [25] using the differential constitutive nonlocal model of Eringen in conjunction with the first-order shear deformation theory. The elastic stability response of curved nanobeams was analyzed by Polit et al. [26] using a nonlocal higher-order shear deformation theory employed in a finite element context. A further application of the nonlocal higher-order theory can be found in the work of Ganapathi and Polit [27] for the numerical study of the bending and buckling response of curved nanobeams, including the thickness stretching effect. For the first time, the shear buckling analysis of porous nanoplates was presented by Shahsavari et al. [28] using a nonlocal quasi-3D plate theory. A different single variable shear deformable nonlocal theory was applied instead, by Shimpi et al. [29], for the static analysis of rectangular micro/nanobeams subjected to a transverse loading, whereas a comprehensive study of the CNTs reinforced composite plates was presented by Karami et al. [30] by applying a nonlocal second-order shear deformable theory.

In a context where curved structures like beams or tubes play a remarkable role in many nanotechnology applications because of their engineering properties (i.e., high strength/stiffness to weight ratios), various size-dependent investigations of reinforced curved beams, tubes, and shells have been carried out in literature [31,32,33,34,35,36,37,38,39,40,41], including different theoretical or computational strategies.

In the current work, the buckling response of CNT reinforced composite curved beams was investigated through the constitutive equations of the nonlocal elasticity, while originally employing the Galerkin method. A continuum model of the nanobeam was also considered based on a higher-order refined theory of beams, which included the shear deformation effects without any proper introduction of shear correction factors. The nonlocal governing equations of the CNT reinforced curved size-dependent beams are here described by means of the Hamiltonian principle, which has been written in a variational form, and they are solved numerically for simply-supported and clamped boundary conditions. After evaluating the accuracy of the proposed method using the available literature, we represent the main results based on a large parametric investigation aimed to studying the influence of boundary conditions, opening angles, CNT distribution patterns, volume fractions, and nonlocal parameters on the critical mechanical buckling force, which is useful for the structural analysis and design of composite curved nanostructures.

The paper is organized as follows. Following the introduction section, we describe the basic fundamentals of the size-dependent problem in Section 2, while the considered solution strategy is presented in Section 3. Afterwards, Section 4 presents the numerical results of a large parametric investigation, useful for design purposes for many engineering applications. Finally, concluding remarks are summarized in Section 5.

## 2. Size-Dependent Problem

### 2.1. Basic Fundamentals

In this section, we consider the nonlocal model of Eringen [20], which is based on the following stress-strain relations:

(1) $$\tau_{ij}=\int_{V}\alpha (\left|x^{\prime }-x\right|),\tau \sigma_{ij}(x^{\prime })d(V^{\prime })$$

$\sigma_{ij}$ and $\tau_{ij}$ being the local and nonlocal stress tensors, together with the following differential equations typically defined for a size-dependent behavior of nanostructure systems:

(2) $$\left(1-{\left(e_{0}a\right)}^{2}\nabla^{2}\right)\sigma_{ij}=C_{ijkl}\varepsilon_{kl}$$

where $\nabla^{2}$ is the Laplacian operator.

**Figure 1:**
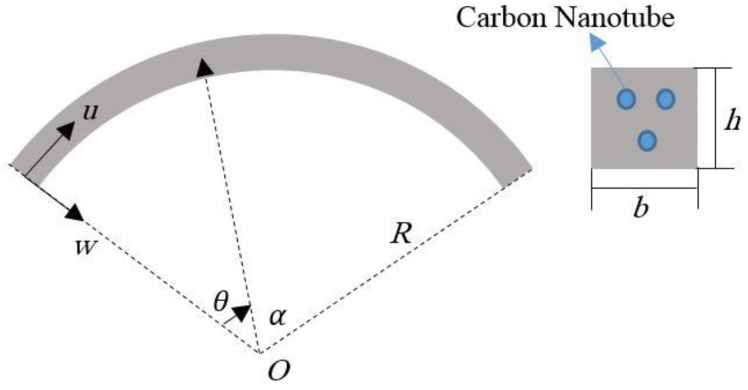
Geometry of a carbon nanotubes (CNTs) reinforced composite curved beam.

**Figure 2:**
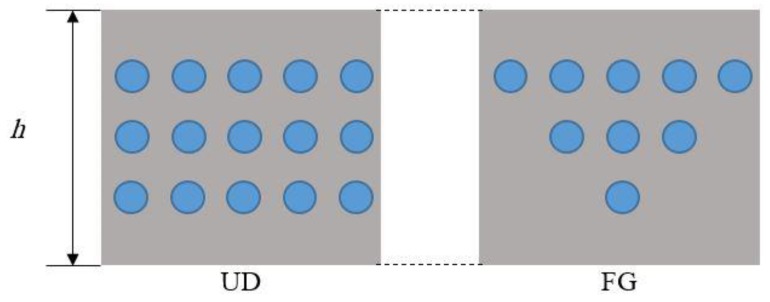
Distribution schemes of CNTs along the thickness direction. UD = uniform distribution; FG = functionally graded.

Let us consider a CNTRC curved beam with length *L* and thickness *h*, as shown in Figure 1. Two different distributions of CNTs are here considered, namely a uniform distribution (UD) and a non-uniform functionally graded (FG) distribution, along the thickness direction of the curved beam (Figure 2), whereby the CNTs are added as filler beside the matrix for the reinforcement purposes. Hence, the effective material properties of CNTRC curved beams are defined, based on the Mori–Tanaka micromechanical scheme and the rule of mixture, as follows [42]:

(3) $$E_{11}=\eta_{1}V_{CNT}E_{11}^{CNT}+V_{m}E^{m}$$

(4) $$\frac{\eta_{2}}{E_{2}}=\frac{V_{CNT}}{E_{22}^{CNT}}+\frac{V_{m}}{E^{m}}$$

(5) $$\frac{\eta_{3}}{G_{12}}=\frac{V_{CNT}}{G_{12}^{CNT}}+\frac{V_{m}}{G^{m}}$$

In the previous relations, $E_{12}^{CNT},E_{22}^{CNT}$ $G_{12}^{CNT}$ are the Young moduli and shear modulus of CNT; *E^m^*, *G^m^* refer to the mechanical properties for the matrix; and $V_{CNT}$ and $V_{m}$ denote the volume fractions of the CNT and matrix, respectively, such that:

(6) $$V_{CNT}+V_{m}=1$$

The CNTs efficiency parameters $\eta_{j}$ in Equations (3)–(5) must be determined before computing the effective material properties of the structure. Thus, we estimate the CNT efficiency parameters $\eta_{1}$ and $\eta_{2}$ by comparing the Young’s moduli $E_{11}^{CNT}$ and $E_{22}^{CNT}$ for the CNTRCs, as obtained by the rule of mixtures, with those given by Han and Elliott [43]. In Table 1, the mechanical properties with a clear good agreement between the molecular dynamics and the rule of mixture are summarized after a proper selection of $\eta_{1}$ and $\eta_{2}$. Moreover, the effective Poisson’s ratio and mass density are expressed as”

(7) $$\nu_{12}=V_{CNT}^{*}\nu_{12}^{CNT}+V_{m}\nu^{m}$$

(8) $$\rho =V_{CNT}\rho^{CNT}+V_{m}\rho^{m}$$

where $\nu_{{}^{12}}^{CNT},\;\rho^{CNT}$ stand for the Poisson’s ratio and mass density of the CNT; and $\nu_{{}^{12}}^{CNT},\;\rho^{CNT}$ refer to the Poisson’s ratio and mass density of the matrix, respectively. The selected distribution schemes for CNTs along the thickness direction can be expressed analytically as [42]:

(9) $$V_{CNT}=\left\{ \begin{array}{ll} V_{CNT}^{*} & \left(\mathrm{UD}\right)\\ \left(1+\frac{2z}{h}\right)V_{CNT}^{*} & \left(\mathrm{FG}\right) \end{array}\right.$$

where:

(10) $$V_{CNT}^{*}=\frac{w_{CNT}}{w_{CNT}+\left(\rho_{CNT}/\rho_{m}\right)-\left(\rho_{CNT}/\rho_{m}\right)w_{CNT}}$$

and $w_{CNT}$ is the mass fraction of the CNTs.

In what follows, we include the interactions among the CNTs and the matrix, while ignoring the effects of strains at general points of the nanocomposite on the stresses at a reference point. Thus, to avoid any possible inaccuracy related to the above-mentioned approximation, it is referred to the presence of nonlocal parameters as required by the Eringen Nonlocal Differential Model (ENDM) to predict the size-dependent behavior of nanostructure systems.

### 2.2. Displacement Field and Strain

According to the refined beam theory, the curved beam is modeled as a continuum model with its displacement field defined as [44]:

(11) $$u_{\theta }(\theta ,r,t)=(1+\frac{z}{R})u(\theta ,t)+\frac{z}{R}(\frac{\partial w_{b}(\theta ,t)}{\partial \theta })+\frac{f(z)}{R}(\frac{\partial w_{s}(\theta ,t)}{\partial \theta })$$

(12) $$w_{r}(\theta ,r,t)=-w_{b}(\theta ,t)-w_{s}(\theta ,t)$$

where *u* is the tangential mid-plane displacement, *w_b_* and *w_s_* are the bending and shear components of the radial displacement, respectively; and *f (z)* is the shape function defined as:

(13) $$f(z)=\frac{\frac{h}{\pi }(\sinh[\frac{\pi z}{h}]-z)}{(\cosh[\frac{\pi }{2}])-1}$$

It is interesting to note that the shape function in Equation (13) satisfies the stress-free boundary conditions on the top and bottom surfaces of the beam without using any shear correction factor. The non-zero strain field related to the displacement components is:

(14) $$\varepsilon_{x}=\varepsilon_{x}^{0}+zk_{x}^{b}+f(z)k_{x}^{s},\;\gamma_{xz}=g(z)(\gamma_{xz}^{0})$$

where:

(15) $$\varepsilon_{x}^{0}=\frac{1}{R}(-w_{b}-w_{s}+\frac{\partial u}{\partial \theta }),\;k_{x}^{b}=\frac{1}{R^{2}}(\frac{\partial u}{\partial \theta }+\frac{\partial^{2}w_{b}}{\partial \theta^{2}}),\;k_{x}^{s}=\frac{f(z)}{R^{2}}(\frac{\partial^{2}w_{s}}{\partial \theta^{2}}),\;\gamma_{xz}^{0}=-\frac{\partial w_{s}}{R\partial \theta }$$

and $g(z)=1f^{\prime }(z)$.

### 2.3. Governing Equations

The equations of motion for the stability of composite curved beams can be derived from the Hamilton’s principle:

(16) $$\int_{0}^{t}\delta (U+V)dt=0$$

where *U* and *V* refer to the strain energy and work done by external forces, respectively. The variational form of the strain energy is expressed as:

(17) $$\begin{array}{l} \delta U=\int_{V}\sigma_{ij}\delta \varepsilon_{ij}dV=\int_{V}(\sigma_{xx}\delta \varepsilon_{xx}+\tau_{xz}\delta \gamma_{xx})dV\\ =\int_{0}^{L}\left(N(-\frac{\delta w_{b}}{R}-\frac{\delta w_{s}}{R}+\frac{\partial \delta u}{R\partial \theta })-\frac{M_{b}}{R^{2}}(\frac{\partial \delta u}{\partial \theta }+\frac{\partial^{2}\delta w_{b}}{\partial \theta^{2}})-\frac{M_{s}}{R^{2}}\frac{\partial^{2}\delta w_{s}}{\partial \theta^{2}}+\frac{Q}{R}\frac{\partial \delta w_{s}}{\partial \theta }\right)Rd\theta \end{array}$$

where:

(18) (N,Mb,Ms)=∫−h2h2(1,z,f(z))σxxdz,  Q=∫−h2h2g(z)τxzdz

Accordingly, the work done by the applied forces takes the following form:

(19) $$\delta V=\int_{0}^{L}\frac{N_{b}}{R^{2}}(\frac{\partial (w_{b}+w_{s})}{\partial \theta }\frac{\partial \delta (w_{b}+w_{s})}{\partial \theta })Rd\theta$$

*N_b_* is the applied tangential force here. By substituting Equations (17), (19) into Equation (16) and integrating by parts with respect to space and time variables, the equations of motion in terms of the displacement components of the curved beam can be obtained as:

(20) $$-\frac{\partial N}{\partial \theta }-\frac{1}{R}\frac{\partial M_{b}}{\partial \theta }=0$$

(21) $$\frac{\partial^{2}M_{b}}{R\partial \theta^{2}}-N-\frac{N_{b}}{R}\frac{\partial^{2}(w_{b}+w_{s})}{\partial \theta^{2}}=0$$

(22) $$\frac{\partial^{2}M_{s}}{R\partial \theta^{2}}-N+\frac{\partial Q}{\partial \theta }-\frac{N_{b}}{R}\frac{\partial^{2}(w_{b}+w_{s})}{\partial \theta^{2}}=0$$

Now, the constitutive equations of the nonlocal refined curved beam are introduced as follows:

(23) $$\sigma_{xx}-\mu \frac{\partial^{2}\sigma_{xx}}{\partial \theta^{2}}=E\varepsilon_{xx}$$

(24) $$\tau_{xz}-\mu \frac{\partial^{2}\tau_{xz}}{\partial \theta^{2}}=G\gamma_{xz}$$

where *µ* = (*e*_0_*a*)^2^. By the combination of Equations (2)–(21), (23), (24), we get to the following relations for the curved beam:

(25) $$N-\mu \frac{\partial^{2}N}{R^{2}\partial \theta^{2}}=\left(\frac{A}{R}\left(-w_{b}-w_{s}+\frac{\partial u}{\partial \theta }\right)+\frac{B}{R^{2}}\left(\frac{\partial u}{\partial \theta }+\frac{\partial^{2}w_{b}}{\partial \theta^{2}}\right)+\frac{B_{s}}{R^{2}}\frac{\partial^{2}w_{s}}{\partial \theta^{2}}\right)$$

(26) $$M_{b}-\mu \frac{\partial^{2}M_{b}}{R^{2}\partial \theta^{2}}=\left(\frac{B}{R}\left(-w_{b}-w_{s}+\frac{\partial u}{\partial \theta }\right)+\frac{D}{R^{2}}\left(\frac{\partial u}{\partial \theta }+\frac{\partial^{2}w_{b}}{\partial \theta^{2}}\right)+\frac{D_{s}}{R^{2}}\frac{\partial^{2}w_{s}}{\partial \theta^{2}}\right)$$

(27) $$M_{s}-\mu \frac{\partial^{2}M_{s}}{R^{2}\partial \theta^{2}}=\left(\frac{B_{s}}{R}\left(-w_{b}-w_{s}+\frac{\partial u}{\partial \theta }\right)+\frac{D_{s}}{R^{2}}\left(\frac{\partial u}{\partial \theta }+\frac{\partial^{2}w_{b}}{\partial \theta^{2}}\right)+\frac{H_{s}}{R^{2}}\frac{\partial^{2}w_{s}}{\partial \theta^{2}}\right)$$

(28) $$Q-\mu \frac{\partial^{2}Q}{R^{2}\partial \theta^{2}}=-\left(\frac{A_{s}}{R}\frac{\partial w_{s}}{\partial \theta }\right)$$

where:

(29) (A,B,Bs,D,Ds,Hs)=∫−h2h2E(1,z,f(z),z2,zf(z),f2(z))dz

(30) As=∫−h2h2g2(z)Gdz

Upon rearrangement, we get to the following governing equations of the beam in terms of displacement components:

(31) $$\begin{array}{l} \frac{A}{R}\left(-\frac{\partial w_{b}}{\partial \theta }-\frac{\partial w_{s}}{\partial \theta }+\frac{\partial^{2}u}{\partial \theta^{2}}\right)+\frac{B}{R^{2}}\left(-\frac{\partial w_{b}}{\partial \theta }-\frac{\partial w_{s}}{\partial \theta }+2\frac{\partial^{2}u}{\partial \theta^{2}}+\frac{\partial^{3}w_{b}}{\partial \theta^{3}}\right)\\ +\frac{B_{s}}{R^{2}}\frac{\partial^{3}w_{s}}{\partial \theta^{3}}+\frac{D}{R^{3}}\left(\frac{\partial^{2}u}{\partial \theta^{2}}+\frac{\partial^{3}w_{b}}{\partial \theta^{3}}\right)+\frac{D_{s}}{R^{3}}\frac{\partial^{3}w_{s}}{\partial \theta^{3}}=0 \end{array}$$

(32) $$\begin{array}{l} \frac{B}{R^{2}}\left(-\frac{\partial^{2}w_{b}}{\partial \theta^{2}}-\frac{\partial^{2}w_{s}}{\partial \theta^{2}}+\frac{\partial^{3}u}{\partial \theta^{3}}\right)+\frac{D}{R^{3}}\left(\frac{\partial^{3}u}{\partial \theta^{3}}+\frac{\partial^{4}w_{b}}{\partial \theta^{4}}\right)+\frac{D_{s}}{R^{3}}\frac{\partial^{4}w_{s}}{\partial \theta^{4}}-\frac{A}{R}\left(-w_{b}-w_{s}+\frac{\partial u}{\partial \theta }\right)\\ -\frac{B}{R^{2}}\left(\frac{\partial^{2}w_{b}}{\partial \theta^{2}}+\frac{\partial u}{\partial \theta }\right)-\frac{B_{s}}{R^{2}}\frac{\partial^{2}w_{s}}{\partial \theta^{2}}-\frac{N_{b}}{R}\frac{\partial^{2}(w_{b}+w_{s})}{\partial \theta^{2}}+\frac{\mu }{R^{2}}\left(\frac{N_{b}}{R}\frac{\partial^{4}(w_{b}+w_{s})}{\partial \theta^{4}}\right)=0 \end{array}$$

(33) $$\begin{array}{l} \frac{B_{s}}{R^{2}}\left(-\frac{\partial^{2}w_{b}}{\partial \theta^{2}}-\frac{\partial^{2}w_{s}}{\partial \theta^{2}}+\frac{\partial^{3}u}{\partial \theta^{3}}\right)+\frac{D_{s}}{R^{3}}\left(\frac{\partial^{3}u}{\partial \theta^{3}}+\frac{\partial^{4}w_{b}}{\partial \theta^{4}}\right)+\frac{H_{s}}{R^{3}}\frac{\partial^{4}w_{s}}{\partial \theta^{4}}\\ -\frac{A}{R}\left(-w_{b}-w_{s}+\frac{\partial u}{\partial \theta }\right)-\frac{B}{R^{2}}\left(\frac{\partial^{2}w_{b}}{\partial \theta^{2}}+\frac{\partial u}{\partial \theta }\right)-\frac{B_{s}}{R^{2}}\frac{\partial^{2}w_{s}}{\partial \theta^{2}}-\frac{A_{s}}{R^{2}}\frac{\partial^{2}w_{s}}{\partial \theta^{2}}\\ -\frac{N_{b}}{R}\frac{\partial^{2}(w_{b}+w_{s})}{\partial \theta^{2}}+\frac{\mu }{R^{2}}\left(\frac{N_{b}}{R}\frac{\partial^{4}(w_{b}+w_{s})}{\partial \theta^{4}}\right)=0 \end{array}$$

## 3. Solution Methodology

The Galerkin method is here employed to solve the equations of motion for functionally graded carbon nanotube-reinforced composite (FG-CNTRC) curved beams with simply-simply (S-S) supports, clamped-simply (C-S) supports, and clamped-clamped (C-C) supports, respectively:

Simply-supports (S):

*w_b_* = *w_s_* = *M* = 0 at *x* = 0, *L*

Clamped-supports (C):

*u* = *w_b_* = *w_s_* = 0 at *x* = 0, *L*

Assuming the following expansion for the displacement field:

(34) $$u(\theta )=\sum_{n=1}^{\infty }U_{n}\frac{\partial F_{m}(\theta )}{\partial \theta }$$

(35) $$w_{b}(\theta )=\sum_{n=1}^{\infty }W_{bn}F_{m}(\theta )$$

(36) $$w_{s}(\theta )=\sum_{n=1}^{\infty }W_{sn}F_{m}(\theta )$$

and by introducing the Equations (34)–(36) into Equations (31)–(33), the following set of relations can be obtained:

(37) $$K\left\{\begin{array}{l} U_{n}\\ W_{bn}\\ W_{sn} \end{array}\right\}=0$$

in which **K** represents the stiffness matrix. The admissible function *F_m_* is selected in the following as the beam eigenfunction, i.e.,

- S-S: $F_{m}=\sin(\frac{n\pi }{\alpha }\theta )$
- C-S: $F_{m}=\sin(\frac{n\pi }{\alpha }\theta )\left[\cos(\frac{n\pi }{\alpha }\theta )-1\right]$
- C-C: $\sin^{2}(\frac{n\pi }{\alpha }\theta )$

To obtain the critical buckling force, we must enforce the determinant of the stiffness matrix equal to zero. This parameter will be quantified in nondimensional form in the next parametric analysis, namely:

(38) $$N_{cr}=N_{b}\frac{R^{2}}{E_{M}h^{3}}$$

## 4. Numerical Results

The procedure proposed in the previous section is here applied to study the size-dependent buckling behavior of FG-CNTRC curved beams. The higher-order shear deformation beam theory is also applied to model the nanobeam, whereby the size-dependent effect is considered by means of the application of the Eringen nonlocal differential model. Thus, the buckling phenomena of the nanostructure are solved mathematically via the Galerkin method for different boundary conditions. The parametric study presented in this work analyzes the sensitivity of the size-dependent buckling response of FG-CNTRC curved beams reinforced with CNTs to some mechanical parameters (i.e., the nonlocal parameter and the nanotube volume fraction), as well as to some geometrical parameters, (namely, the opening angle, slenderness ratio, and the CNT distribution schemes). The preliminary focus of the investigation was on the accuracy of the proposed method to compute the critical buckling load, whose results are summarized in Table 2 in nondimensional form for an S-S beam, while varying the nonlocal parameter µ. Based on a comparative evaluation between our predictions and those obtained by Reddy [45], Aydogdu [46], and Eltaher [47], a very good match was observed, which confirms the accuracy of the proposed formulation for similar problems.

**Figure 3:**
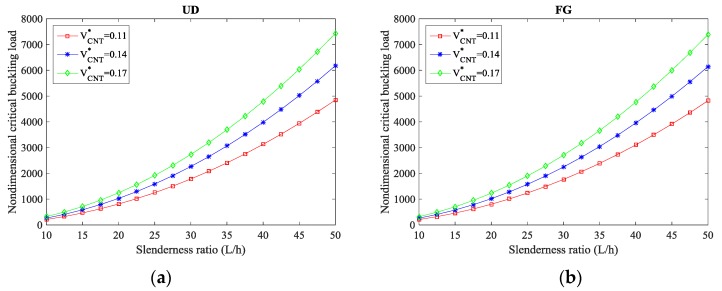
Critical buckling load versus slenderness ratio for different volume fractions and distribution patterns of CNTs: (**a**) UD pattern, (**b**) FG pattern. (µ = 2 nm^2^, *L* = 20, α = π/3).

**Figure 4:**
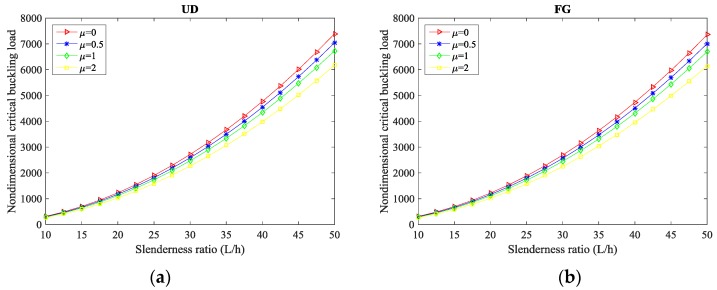
Critical buckling load versus slenderness ratio for different nonlocal parameters and distribution patterns of CNTs: (**a**) UD pattern, (**b**) FG pattern. (*L* = 20, α = π/3, $V_{CNT}^{*}$ = 0.14).

**Figure 5:**
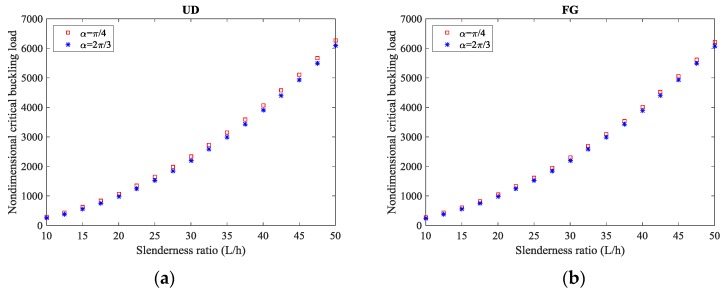
Critical buckling load versus slenderness ratio for different opening angles and distribution patterns of CNTs: (**a**) UD pattern, (**b**) FG pattern. (µ = 1 nm^2^, *L* = 20, $V_{CNT}^{*}$ = 0.14).

Next, we discuss about the size-dependence of the buckling load for FG-CNTRC curved beams with different boundary conditions (see Table 3, Table 4, Table 5, Table 6, Table 7, Table 8, Table 9, Table 10 and Table 11 and Figure 3, Figure 4 and Figure 5), together with results for UD-CNTRC counterparts, for a direct comparison. Unless otherwise stated before, the length of the curved beam is fixed at *L* = 20, whereby a Poly{(m-phenylenevinylene)-co-[(2,5-dioctoxy-p-phenylene) vinylene]} is selected as matrix (henceforth labeled as PmPV), with Poisson’s ratio $\nu^{m}=0.34$, elastic modulus $E^{m}=2.1\;\mathrm{GPa}$, and temperature T = 300 K. As reinforcement phase, instead, we select an armchair (10, 10) SWCNTs, with elastic moduli $E_{11}^{CNT}=5.6466\;\mathrm{TPa}$, $E_{22}^{CNT}=7.080\;\mathrm{TPa}$, and Poisson’s ratio $\nu^{CNT}=0.175$.

More specifically, Table 3, Table 4 and Table 5 evaluate the effect of the volume fraction and distribution patterns of CNTs on the nondimensional critical buckling load of the composite curved beams for S-S, C-S, and C-C CNTRC curved beams, respectively, while *L/h* = 1 and α = π/3 are considered. By exploiting the numerical results in Table 3, Table 4 and Table 5 comparatively, it is worth noting that clamped nanostructures yield the maximum buckling load, while S-S beams get the lowest buckling values. Moreover, an increment in the volume fraction of CNTs $V_{CNT}^{*}$ significantly raises the buckling load of both UD- and FG-CNTRCs, with its behavior also affected by the nonlocality µ. More specifically, a rise in nonlocality reduces the buckling load of CNTRC curved beams because of the stiffness-softening mechanisms characterizing the nanostructure. The sensitivity of the buckling response to the volume fraction of CNTs is also plotted in Figure 3 versus the slenderness ratio *L/h*, for a C-C boundary condition and different distributions of CNTs (namely a UD pattern in Figure 3a and an FG pattern in Figure 3b). Based on Figure 3, it is worth to note that the monotone behavior of the critical buckling load increases for development in slenderness ratios *L/h*, especially for the higher values of the volume fraction of CNTs $V_{CNT}^{*}$.

In addition, Table 6, Table 7 and Table 8 summarize the results of the nondimensional critical buckling load for different *L/h* ratios and nonlocal parameters µ, while considering a S-S, C-S, and C-C composite curved beams reinforced with CNTs, respectively. It is clear that the highest sensitivity of the buckling response of curved beams to the length-to-thickness ratio is obtained for C-C boundary conditions, followed by C-S, and S-S supports, respectively. Moreover, the highest value of the critical load is always reached in size-dependent composite curved beams with µ = 0, whereby as µ increases, the buckling load decreases, independently of the selected *L/h* ratios and CNTs distributions. A meaningful sensitivity of the response to the boundary conditions is also detected due to an expectable variation in the structural stiffness of the composite curved beams. Furthermore, Figure 4 illustrates the double effect of the nonlocal parameter and the slenderness ratio *L/h* on the nondimensional critical buckling load of CNTRC curved beams for fixed C-C boundary conditions and different CNTs distribution patterns (namely, a UD pattern in Figure 4a and an FG pattern in Figure 4b).

It is worth noting that the moderately thick CNTRC curved beam with *L/h* = 10 features the lowest critical buckling load. This last one increases as the length-to-thickness ratio *L/h* is increased, both in UD and FG-CNTRC curved beams. Another key aspect related to the sensitivity of the response with the nonlocal parameter is that the impact is more pronounced for higher values of *L/h*, (or equivalently to a lower thickness of the curved beam for a fixed length).

The effect of the opening angle and the nonlocal parameter on the nondimensional critical buckling load is shown in Table 9, Table 10 and Table 11 for S-S, C-S, and C-C CNTs reinforced composite curved beams, respectively. By exploiting comparatively, the results can be found that an increasing value of the opening angle decreases the buckling load whose value is also affected by the selected boundary condition. The results are obtained far from a size-dependence of the structure. It means that the buckling load of size-dependent and independent response of curved beams decreases by increasing the opening angle for each boundary conditions.

The double effect of the opening angle and slenderness ratio is finally emphasized in Figure 5 for each CNT reinforcement patterns, while considering a fixed C-C boundary condition. Based on this last plot, it is clearly visible that the higher sensitivity of the response for thick CNTs reinforced curved beams (i.e., for *L/h* = 50) compared to thin structures. 

## 5. Conclusions

The size-dependent buckling of FG-CNTRC curved beams was investigated within the framework of a refined beam theory and Eringen nonlocal differential model. The CNTs distributions were considered uniform and graded through the thickness direction, and the material properties were estimated using the rule of mixtures. The Galerkin method was also employed to obtain the critical buckling load of FG-CNTRC curved beams for different boundary conditions. The effects of the nonlocal parameter, CNT volume fraction, slenderness ratio, opening angle, boundary conditions, and CNTs distribution scheme on the critical buckling load of FG-CNTRC curved beams were discussed in detail. Based on the numerical results, the following concluding remarks can be summarized: 

An increase in CNT volume fraction leads to an increase in the critical buckling load for both UD- and FG-CNTRC curved beams.

A UD of CNTs in composite curved beams yields higher values of the critical buckling load compared to an FG distribution of CNTs. 

An increase in the opening angle leads to a lower value of the critical buckling load for both UD- and FG-CNTRC curved beams.

The highest values of the critical buckling load of FG-CNTRC curved beams is obtained for completely clamped C-C boundary conditions, due to an increase in structural stiffness compared to simply supported boundary conditions. 

Using nonlocality phenomena, the critical buckling load of FG-CNTRC curved beam decreases. Moreover, the effect of the nonlocal parameter in curved beams with higher slenderness ratios is more pronounced, if compared to lower slenderness ratios.

## Figures and Tables

**Table 1:** Mechanical properties for a Poly{(m-phenylenevinylene)-co-[(2,5-dioctoxy-p-phenylene) vinylene]} (PmPV)/CNT composites reinforced by (10,10) SWCNT at the temperature T = 300 K.

$V_{CNT}^{*}$	MD [43]		Rule of Mixture			
	E_11_ (GPa)	E_22_ (GPa)	E_11_ (GPa)	η_1_	E_22_ (GPa)	η_2_
0.11	94.8	2.2	94.42	0.149	2.20	0.934
0.14	120.2	2.3	120.38	0.150	2.30	0.941
0.17	145.6	3.5	144.77	0.140	3.49	1.381

**Table 2:** Nondimensional buckling force for a simply supported beam.

µ	Reddy [45]	Aydogdu [46]	Eltaher [47]	Present
0	9.8696	9.8696	9.86973	9.80601
1	8.9830	9.6319	8.98312	8.92692
2	8.2426	9.4055	8.24267	8.19176
3	7.6149	9.1894	7.61499	7.56846
4	7.0761	8.9830	7.07614	7.03246

**Table 3:** Nondimensional critical buckling load for simply- simply (S-S) CNTRC curved beams with *L/h* = 10, α = π/3.

	$V_{CNT}^{*}$	µ = 0	µ = 0.5	µ = 1	µ = 1.5	µ = 2	µ = 3
UD-CNTRC	0.11	12.5642	12.4111	12.2617	12.1158	11.9734	11.6983
	0.14	14.3533	14.1784	14.0077	13.8411	13.6783	13.3641
	0.17	19.6015	19.3626	19.1295	18.9019	18.6797	18.2505
FG-CNTRC	0.11	10.1067	9.9835	9.8633	9.7459	9.6314	9.4101
	0.14	11.7678	11.6244	11.4844	11.3478	11.2144	10.9567
	0.17	15.6689	15.4780	15.2916	15.1097	14.9321	14.5890

**Table 4:** Nondimensional critical buckling load for clamped simply (C-S) CNTRC curved beams with *L/h* = 10, α = π/3.

	$V_{CNT}^{*}$	µ = 0	µ = 0.5	µ = 1	µ = 1.5	µ = 2	µ = 3
UD-CNTRC	0.11	123.0005	119.3204	115.8541	112.5835	109.4924	103.7931
	0.14	153.2617	148.6762	144.3571	140.2818	136.4303	129.3288
	0.17	189.3549	183.6894	178.3532	173.3182	168.5597	159.7857
FG-CNTRC	0.11	120.5401	116.9336	113.5366	110.3314	107.3022	101.7169
	0.14	150.8821	146.3677	142.1157	138.1037	134.3120	127.3207
	0.17	185.4017	179.8545	174.6296	169.6998	165.0406	156.4498

**Table 5:** Nondimensional critical buckling load for clamped- clamped (C-C) CNTRC curved beams with *L/h* = 10, α = π/3.

	$V_{CNT}^{*}$	µ = 0	µ = 0.5	µ = 1	µ = 1.5	µ = 2	µ = 3
UD-CNTRC	0.11	251.6676	239.8324	229.0603	219.2143	210.1798	194.1748
	0.14	316.4206	301.5402	287.9965	275.6171	264.2581	244.1351
	0.17	386.8498	368.6573	352.0990	336.9642	323.0770	298.4749
FG-CNTRC	0.11	249.5883	237.8509	227.1678	217.4031	208.4433	192.5705
	0.14	314.4762	299.6872	286.2267	273.9234	262.6343	242.6349
	0.17	383.5269	365.4907	349.0746	334.0698	320.3018	295.9111

**Table 6:** Effect of the slenderness ratio *L/h* on the nondimensional critical buckling load for S-S CNTRC curved beams with α = π/3, $V_{CNT}^{*}$ = 0.14.

	*L/h*	µ = 0	µ = 0.5	µ = 1	µ = 1.5	µ = 2	µ = 3
UD-CNTRC	10	14.3533	14.1784	14.0077	13.8411	13.6783	13.3641
	20	25.2537	24.9459	24.6456	24.3524	24.0660	23.5132
	30	29.4450	29.0862	28.7360	28.3941	28.0603	27.4156
	50	32.1881	31.7958	31.4130	31.0393	30.6744	29.9697
FG-CNTRC	10	11.7678	11.6244	11.4844	11.3478	11.2144	10.9567
	20	18.4256	18.2010	17.9819	17.7680	17.5591	17.1557
	30	20.6574	20.4057	20.1600	19.9202	19.6860	19.2337
	50	22.0620	21.7931	21.5307	21.2746	21.0244	20.5414

**Table 7:** Effect of the slenderness ratio *L/h* on the nondimensional critical buckling load for C-S CNTRC curved beams with α = π/3, $V_{CNT}^{*}$ = 0.14.

	*L/h*	µ = 0	µ = 0.5	µ = 1	µ = 1.5	µ = 2	µ = 3
UD-CNTRC	10	153.2617	148.6762	144.3571	140.2818	136.4303	129.3288
	20	575.5663	558.3455	542.1252	526.8208	512.3568	485.6873
	30	1247.1406	1209.8265	1174.6804	1141.5187	1110.1779	1052.3904
	50	3353.4099	3253.0768	3158.5732	3069.4054	2985.1338	2829.7502
FG-CNTRC	10	150.8821	146.3677	142.1157	138.1037	134.3120	127.3207
	20	564.4312	547.5436	531.6372	516.6288	502.4446	476.2911
	30	1228.5852	1191.8263	1157.2031	1124.5348	1093.6603	1036.7326
	50	3327.6844	3228.1210	3134.3424	3045.8586	2962.2334	2808.0419

**Table 8:** Effect of the slenderness ratio *L/h* on the nondimensional critical buckling load for C-C CNTRC curved beams with α = π/3, $V_{CNT}^{*}$ = 0.14.

	*L/h*	µ = 0	µ = 0.5	µ = 1	µ = 1.5	µ = 2	µ = 3
UD-CNTRC	10	316.4206	301.5402	287.9965	275.6171	264.2581	244.1351
	20	1230.3705	1172.5095	1119.8461	1071.7102	1027.5419	949.2954
	30	2714.6098	2586.9490	2470.7560	2364.5519	2267.1018	2094.4639
	50	7393.5474	7045.8487	6729.3838	6440.1251	6174.7087	5704.5098
FG-CNTRC	10	314.4762	299.6872	286.2267	273.9234	262.6343	242.6349
	20	1218.4960	1161.1934	1109.0383	1061.3669	1017.6249	940.1336
	30	2691.1353	2564.5784	2449.3901	2344.1045	2247.4971	2076.3521
	50	7355.7738	7009.8515	6695.0035	6407.2225	6143.1622	5675.3655

**Table 9:** Effect of the opening angle α on the nondimensional critical buckling load for S-S CNTRC curved beams with *L/h* = 10, $V_{CNT}^{*}$ = 0.14.

	*α*	µ = 0	µ = 0.5	µ = 1	µ = 1.5	µ = 2	µ = 3
UD-CNTRC	π/4	28.3891	28.0431	27.7055	27.3759	27.0540	26.4325
	π/3	14.3533	14.1784	14.0077	13.8411	13.6783	13.3641
	π/2	4.5393	4.4840	4.4300	4.3773	4.3258	4.2264
	2π/3	1.4001	1.3830	1.3664	1.3501	1.3342	1.3036
FG-CNTRC	π/4	23.3784	23.0935	22.8155	22.5440	22.2790	21.7672
	π/3	11.7678	11.6244	11.4844	11.3478	11.2144	10.9567
	π/2	3.6889	3.6440	3.6001	3.5573	3.5155	3.4347
	2π/3	1.1279	1.1141	1.1007	1.0876	1.0748	1.0501

**Table 10:** Effect of the opening angle α on the nondimensional critical buckling load for C-S CNTRC curved beams with *L/h* = 10, $V_{CNT}^{*}$ = 0.14.

	*α*	µ = 0	µ = 0.5	µ = 1	µ = 1.5	µ = 2	µ = 3
UD-CNTRC	π/4	172.4731	167.3128	162.4523	157.8662	153.5319	145.5402
	π/3	153.2617	148.6762	144.3571	140.2818	136.4303	129.3288
	π/2	139.5855	135.4092	131.4754	127.7638	124.2560	117.7882
	2π/3	134.8634	130.8284	127.0277	123.4417	120.0526	113.8035
FG-CNTRC	π/4	168.1669	163.1354	158.3962	153.9246	149.6986	141.9064
	π/3	150.8821	146.3677	142.1157	138.1037	134.3120	127.3207
	π/2	138.6057	134.4586	130.5525	126.8670	123.3838	116.9614
	2π/3	134.3841	130.3633	126.5762	123.0029	119.6258	113.3990

**Table 11:** Effect of the opening angle α on the nondimensional critical buckling load for C-C CNTRC curved beams with *L/h* = 10, $V_{CNT}^{*}$ = 0.14.

	*α*	µ = 0	µ = 0.5	µ = 1	µ = 1.5	µ = 2	µ = 3
UD-CNTRC	π/4	337.8116	321.9253	307.4660	294.2497	282.1228	260.6394
	π/3	316.4206	301.5402	287.9965	275.6171	264.2581	244.1351
	π/2	301.1614	286.9986	274.1080	262.3256	251.5144	232.3618
	2π/3	295.8488	281.9358	269.2726	257.6981	247.0776	228.2628
FG-CNTRC	π/4	334.3510	318.6274	304.3162	291.2353	279.2327	257.9693
	π/3	314.4762	299.6872	286.2267	273.9234	262.6343	242.6349
	π/2	300.3202	286.1970	273.3424	261.5930	250.8119	231.7128
	2π/3	295.4045	281.5124	268.8683	257.3111	246.7066	227.9201
